# Cavernous transformation of the portal vein in pancreatic cancer surgery—venous bypass graft first

**DOI:** 10.1007/s00423-020-01974-0

**Published:** 2020-09-11

**Authors:** Thomas Schmidt, Oliver Strobel, Martin Schneider, Markus K. Diener, Christoph Berchtold, André L. Mihaljevic, Arianeb Mehrabi, Beat P. Müller-Stich, Thilo Hackert, Markus W. Büchler

**Affiliations:** Department of General, Visceral and Transplantation Surgery, University of Heidelberg, Im Neuenheimer Feld 110, 69120 Heidelberg, Germany

**Keywords:** Pancreatic cancer, Surgery, Cavernous transformation, Venous bypass graft

## Abstract

**Background:**

In recent years, several techniques have been introduced to allow safe oncologic resections of cancers of the pancreatic head. While resections of the mesenterico-portal axis became now a part of the routine treatment, patients with a cavernous transformation of the portal vein still pose a surgical challenge and are regularly deemed unresectable.

**Objective:**

Here, we describe a technique of initial venous bypass graft placement between the superior mesenteric vein or its tributaries and the portal vein before the resection of the pancreatic head. This approach avoids uncontrollable bleeding as well as venous congestion of the intestine with a continuous hepatic perfusion and facilitates oncologic resection of pancreatic head cancers. This technique, in combination with previously published resection strategies, enables tumor resection in locally advanced pancreatic head cancers.

**Conclusions:**

Venous bypass graft first operations facilitate and enable the resection of the pancreatic head cancers in patients with a cavernous transformation of the portal vein thus rendering these patients resectable.

## Introduction

Pancreatic cancer is the fourth leading cause of cancer-related death in the Western world and still has a rising incidence [1]. Despite recent advances in systemic chemotherapy, long-term survival and cure are only possible with complete surgical resection [2, 3]. In initially unresectable or borderline resectable cases, a complete tumor resection can be achieved after neoadjuvant chemotherapy or chemoradiation, especially after the introduction of the FOLFIRINOX regimen [4–7]. Even in locally advanced cases with tumor infiltration into the mesenterico-portal axis (MPA), the celiac artery (CA), the hepatic arteries, or the superior mesenteric artery (SMA) as well as surrounding organs, an extended resection can be warranted in selected cases [8–12]. To achieve complete oncologic resections of tumors in the pancreatic head, several surgical modifications to conventional partial pancreatoduodenectomy (PD) have been introduced in the last decade. The aim of these meanwhile standardized approaches is both an increase in safety as well as oncological radicality. These standardized approaches include “uncinate process first,” “artery first,” and the “triangle operation” which have been described recently [13–17]. The combination of these techniques allows a systematic mesopancreatic dissection along the SMA together with the dissection alongside the CA, with a standardized radical clearance of potentially tumor-infiltrated lymphatic and neural tissue [15, 18].

While a MPA resection with reconstruction is nowadays a standard procedure for tumors with a portal vein (PV) or superior mesenteric vein (SMV) infiltration, a cavernous transformation of the portal vein as a consequence of a complete portal vein occlusion still remains a dreaded challenge in pancreatic surgery [11, 19–22]. In patients with a cavernous transformation, a pancreatic head resection can cause life-threatening intraoperative venous bleeding as well as a congestion of the intestine. Here, we provide a step-by-step instruction for pancreatic head resections with a “venous bypass graft first” approach for patients with cavernous transformation.

## Surgical technique

### Preoperative assessment of the superior mesenteric and portal vein

**Fig. 1:**
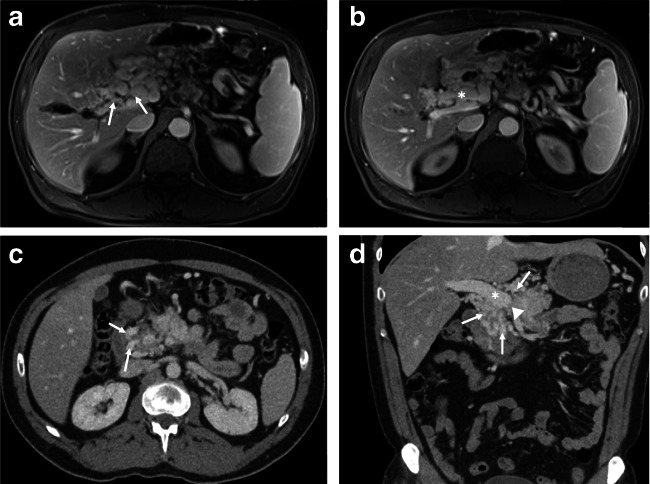
*Preoperative imaging.* **a** Preoperative MRI imaging of a patient with pancreatic cancer indicating a cavernous transformation of the portal vein in the hepatoduodenal ligament (indicated with arrows). **b** Patent extrahepatic portal vein in the same patients as in (**a**) (indicated by an asterisk). **c**, **d** Contrast-enhanced CT imaging of a patient with a pancreatic head adenocarcinoma and cavernous transformation of the portal vein (cavernous transformation indicated with arrows, patent extrahepatic portal vein (asterisk), site of portal vein occlusion (arrowhead))

To assess the possibility of a “venous bypass graft first” operation in patients with a cavernous transformation of the MPA after complete occlusion of the splenic-mesenteric confluence, the anatomy of the PV and the SMV has to be carefully analyzed in preoperative contrast-enhanced CT or MRI scans (Fig. 1a–d). It is quintessential to have a patent albeit short extrahepatic portal vein as well as a patent SMV or in case of tumor infiltration into the mesentery, a large tributary branch of the SMV proximal to the occlusion to enable a venous bypass using a graft; therefore, preoperatively, a thrombosis of or occlusion of the extrahepatic portal vein needs to be ruled out.

### Exploration and identification of venous vessels for a graft

**Fig. 2:**
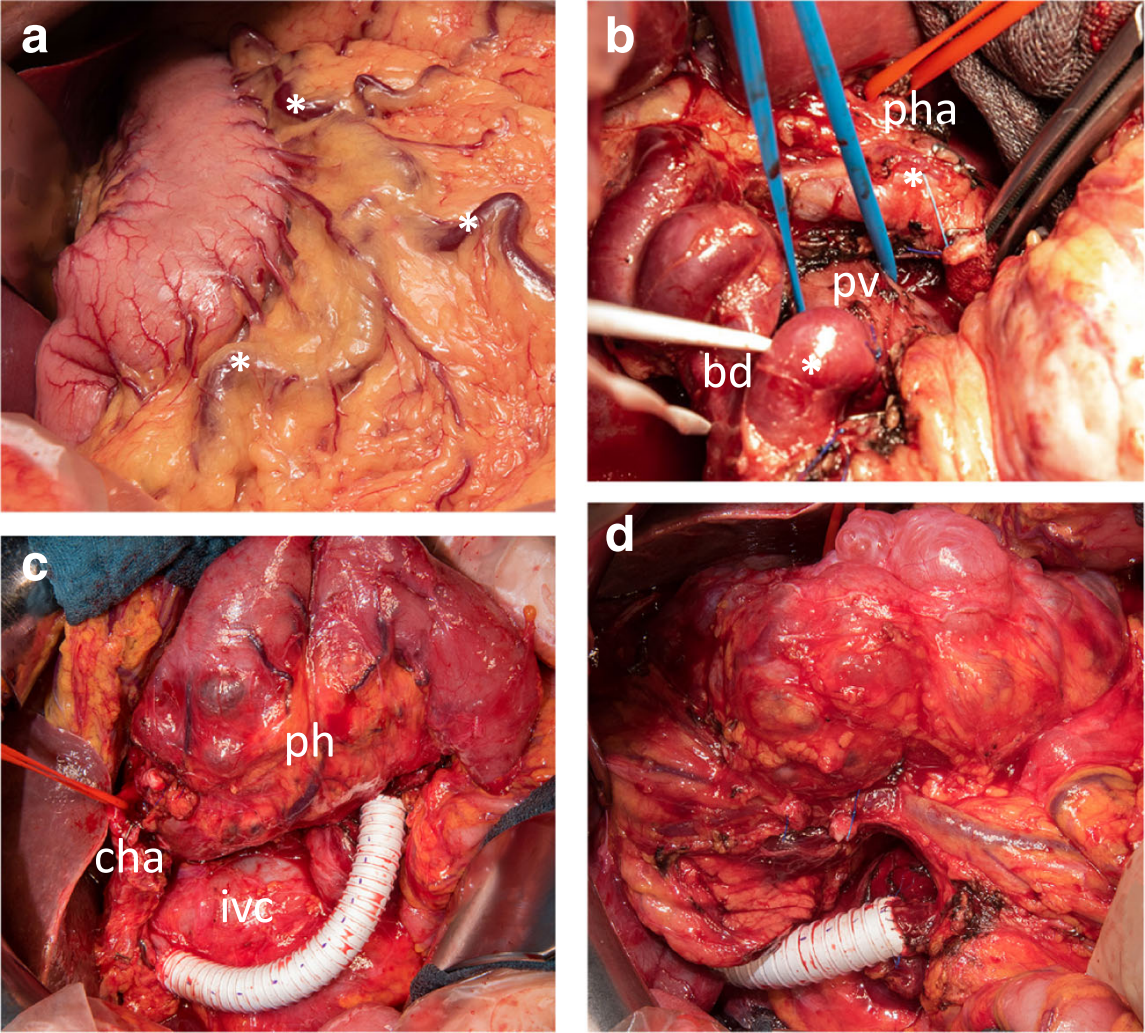
Venous graft first placement. **a** Venous congestion and portal hypertension observed upon initial explorative laparotomy. An asterisk indicates congested varicose veins alongside the stomach and in the greater omentum due to portal hypertension. **b** Exploration and dissection of the hepatoduodenal ligament. Bile duct (bd) is tagged with a white vessel loop, the extrahepatic portal vein (pv) is tagged with a blue vessel loop, and the proper hepatic artery (pha) with a red vessel loop. **c**, **d** 12-mm ring-enforced polytetrafluoroethylene Gore-Tex prosthesis as venous bypass graft before pancreatic head (ph) resection between superior mesenteric vein and portal vein. Placement of the graft behind the ph in (**d**). cha, common hepatic artery

The operation begins with the initial exploration as in a conventional partial pancreaticoduodenectomy. High blood loss due to the cavernous transformation with excessive varicose veins should be avoided. During the exploration phase, excessive varicose veins can be visible around the stomach and in the greater omentum (Fig. 2a). After separation of the greater omentum from the transverse colon, the right colic flexure is mobilized to gain access to the head of the pancreas. To provide sufficient access and flexibility of the mesenteric root, the complete right hemicolon and the mesenteric root can be mobilized. The aim in the first stage of the operation is to identify suitable venous vessels to perform the “venous bypass graft first” approach. This includes the identification of the superior mesenteric vein or one of its branches as well as the portal vein in the hepatoduodenal ligament, often close to the liver hilum. A potential vein for the proximal intestinal part of the bypass should be secured with a vessel loop. This might be the superior mesenteric vein itself or one or more of its dilated tributaries. To assess the resectability of the tumor, an extended Kocher’s maneuver is performed. This includes the identification of the left renal vein and its junction with the inferior vena cava. Additionally, the root of the superior mesenteric artery should be secured at its aortic origin [15, 16]. In the next step, the dissection of the hepatoduodenal ligament is performed (Fig. 2b). If possible, the proper hepatic artery with the left and right hepatic arteries is identified and secured with vessel loops. After securing arterial inflow control to the liver, the extrahepatic portal vein close to the liver hilum should be secured with a vessel loop as this serves as the distal anastomotic location of the venous bypass. The common bile duct with its varicose transformed veins can be a challenge when accessing the extrahepatic portal vein**.**

### Venous graft first approach

The superior mesenteric vein or a large branch is transected after a vascular clamp is applied. The pancreatic side of the SMV is suture ligated and, on the mesenteric side, an end-to-end anastomosis is performed (i.e., 5–0 monofilament non-resorbable running suture) with a ring-enforced polytetrafluoroethylene (PTFE) Gore-Tex prosthesis of 10 or 12 mm diameter depending on the SMV or SMV branch size. The prosthesis is placed around the dorsal part of the pancreatic head with a sufficient length to enable further resection of the tumor. The portal vein is transected after the application of vascular clamps and a suture ligation towards the pancreatic head is placed. The prosthesis is flushed with heparinized saline solution and an end-to-end anastomosis is performed to the portal vein in the liver hilum (Fig. 2c, d). After the release of the vascular clamps, flow is measured and the intestine is observed for venous congestion. Postoperatively, a standard thrombosis prophylaxis is performed with low molecular weight heparin.

### Resection after venous graft placement

The common hepatic artery is followed towards the CA and the gastroduodenal artery is identified. The gastroduodenal artery is severed as well as the right gastroepiploic vessels. The distal stomach or duodenum (pylorus-preserving PD) is transected and the remnant stomach is placed in the left upper quadrant. During this phase of the operation, the left gastric vein is usually severed due to portal vein infiltration. The left gastric vein is tagged for potential re-anastomosis. The jejunum is transected 10 cm after the ligament of Treitz. The proximal jejunum is devascularized and transposed via the opened ligament of Treitz into the right upper quadrant. Afterward, the uncinate process is mobilized from the superior mesenteric artery analogous to the uncinate first approach [14].

To perform the portal vein resection, the pancreas is divided left to the normal resection plane of the SMV. The splenic artery is identified and preserved at its location at the superior border of the pancreas. After the division of the pancreas, the splenic vein is identified and divided. During the resection phase, the dissection of the pancreatic head is performed according to the previously published “artery first” and “uncinate” approaches [14, 16]. The vascular approach during this phase of the operation was described previously [15]. Finally, the pancreatic head is resected in a right to left and posterior to anterior fashion and the mesopancreas is resected up to the transection plane of the pancreas. For a complete oncologic resection, we perform additionally a resection of the potentially tumor-infiltrated lymph node and neural tissue in the triangular space between the CA, aorta, and SMA [15]. The anterior border of the “triangle” consists usually of the portal vein, but is resected in this approach. Due to the extensive tumor infiltration, total pancreatectomy is often warranted. To relieve the gastric congestion, the left gastric vein, or the right gastroepiploic vein, can be implanted into the left renal vein. Alternatively, the splenic vein can be implanted into the left renal vein. If these approaches are not possible, a subtotal gastrectomy should be considered.

### Shortening or removal of the venous bypass

**Fig. 3:**
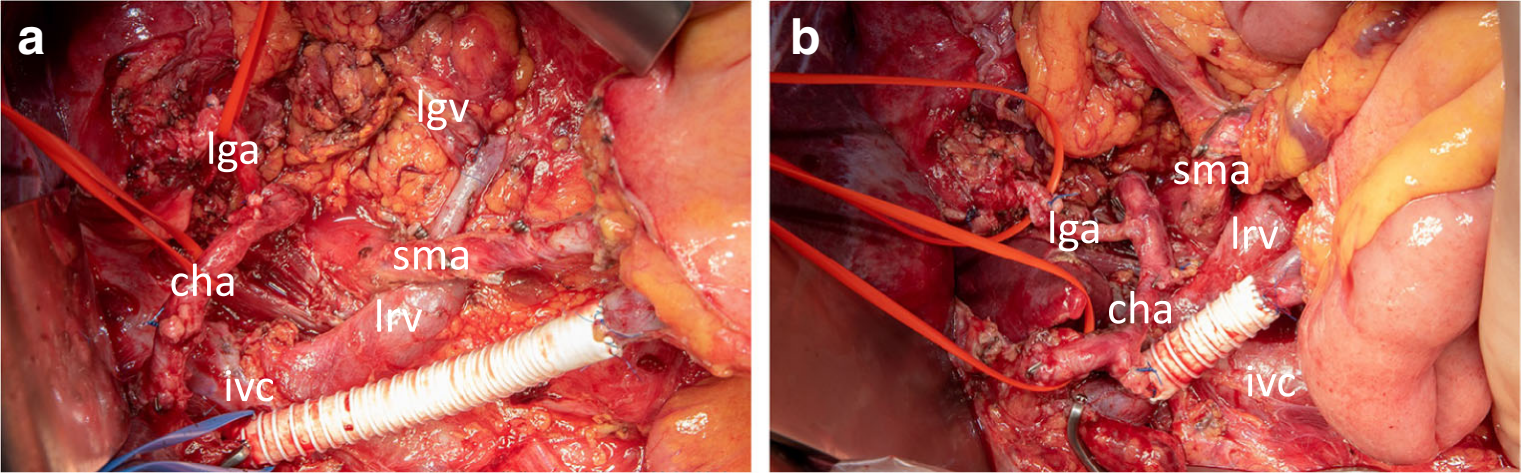
Extended resection and venous bypass graft shortening. **a** Intraoperative situs after total pancreatectomy. Venous bypass graft placed between superior mesenteric vein (smv) and portal vein. Anastomosis between left gastric vein (lgv) and left renal vein (lrv) to relief left-sided portal hypertension. Common hepatic artery (cha) and left gastric artery (lga) are tagged with vessel loops. Complete “triangle” lymphadenectomy between superior mesenteric artery (sma) and celiac trunk. **b** Shortening of the venous bypass graft before reconstruction to avoid kinking of the graft, pv, and smv as well as the sma. ivc, inferior vena cava

Before reconstruction, the venous bypass graft should be shortened to the correct length. This is important to avoid graft kinking and venous thrombosis. In some cases, the complete graft can be removed if an end-to-end anastomosis of the SMV and PV is possible; however, this is limited by potential kinking of the SMA, which needs to be avoided. The shortening of the venous graft completes the resection phase of the operation. To avoid left-sided portal hypertension, a splenorenal shunt or a shunt from the left gastric vein to the left renal vein should be considered (Fig. 3a, b).

## Discussion

Here, we describe an approach to render patients with a cavernous transformation of the portal vein due to occlusion of the splenic-mesenteric confluence by pancreatic head adenocarcinomas resectable in a controlled way avoiding fatal bleeding complications.

While tumor infiltration into the portal vein or superior mesenteric vein is no longer a contraindication for pancreatic head resection in pancreatic adenocarcinoma [8, 10, 11, 18, 20, 21, 23, 24], the complete occlusion of the mesenterico-portal vein with a cavernous transformation remains a challenge. This challenge is both of technical and oncologic nature. Due to the varicose transformations of the collateral veins around the pancreas, the dissection of the pancreatic head can lead to massive bleeding. Controlling this bleeding by dissecting the veins may lead to an intolerable congestion of the intestines. Additionally, the hepatic perfusion can be limited when the venous collaterals are severed. All these intraoperative challenges can lead to additional postoperative morbidity and mortality. Oncologically, the challenge consists of large and advanced tumors; however, a R0 resection should remain the main objective of surgical resection [2, 3]. The oncologic outcome in initially unresectable or borderline resectable patients can be improved by neoadjuvant therapy rendering more of these patients potentially resectable [4, 25, 26]. Due to this, more patients are presented to the surgeons and new surgical approaches are warranted.

In the past years, many strategies were discussed to alleviate left-sided portal hypertension or mesenteric hypertension with cavernous transformation of the portal vein. Mesenteric venous shunts were initially developed to treat gastroesophageal variceal hemorrhage in patients with end-stage liver disease and portal hypertension. Now different versions of these shunts are also employed in pancreatic cancer surgery often developed from techniques used for extended pancreatic resections [27, 28]. This can include temporary mesocaval shunts, which can be utilized to temporarily divert portal flow allowing for a safe portal dissection [29, 30]. Also, the SMV can be directly sewn to the inferior vena cava (IVC) or an interposition graft can be utilized [31]. For the interposition graft, it was described that autologous veins can be used. This includes for example the internal jugular vein to establish a temporary mesocaval shunt between the SMV and the IVC described by different groups [32, 33]. An additional challenge in this case persists if the inferior mesenteric vein does not provide retrograde decompression of the left-sided portal hypertension. In these cases, another shunt from the left side with for example a splenorenal shunt is needed adding to the complexity of the procedure [29]. All the approaches limit the blood flow through the liver during the time of the resection for up to several hours potentially leading even to portal vein thrombosis limiting their application.

Portal vein occlusion with extensive collateral vein formation is also a challenge in the surgery of patients with chronic pancreatitis for which different surgical techniques were developed [34, 35]. The introduced methods specifically avoid dissection of the pancreas from the portal and superior mesenteric vein. In the multicenter randomized controlled ChroPac trial, it was concluded that in patients with a compression or occlusion of the portal vein system, a duodenum-preserving pancreatic head resection should be the procedure of choice instead of a partial pancreatoduodenectomy [36]. Our approach could render these patients accessible to a partial pancreatoduodenectomy, which might be warranted as 5% of all patients in the trial had an incidental detection of pancreatic cancer [36].

Our described approach provides continuous portal blood flow to the liver throughout the resection and reconstruction phase of the operation. In approaches that propose a mesocaval shunt, this is a limitation that cannot be avoided. Latest upon transection of the bile duct, the remaining blow flow via the cavernous transformed collateral veins is stopped. This leads inevitably to a prolonged liver ischemia/low perfusion which is of special importance as many patients already underwent multiple cycles of neoadjuvant hepatotoxic chemotherapy. Potential arterial clamping of the CA during the resection phase leads to a complete hepatic ischemia in approaches with a mesocaval shunt. However, rather than comparing our technique to alternative approaches which were highlighted, this article intends to provide an additional solution for a remaining challenge for pancreatic surgeons. Also, previously, Bachellier and colleagues suggested a similar surgical approach and commented on the advantages [37]. In their manuscript, they reported that this procedure is safe as no postoperative mortality occurred. Often in this procedure also, an arterial resection was warranted. In 15 patients, the authors analyzed an overall mean survival of 17 months with a 3-year survival of 11% was reported [37].

Surgical approaches which render unresectable patients amenable to radical local tumor resection are of high value for this small but existing subgroup of patients with pancreatic cancer suffering locally advanced but non-metastasized tumors. After neoadjuvant therapy, surgical resection becomes feasible in up to 60% of PDAC patients, which in the future could include more patients with cavernous transformed portal veins [18, 38].
